# The Proteasome Activator PA28γ, a Negative Regulator of p53, Is Transcriptionally Up-Regulated by p53

**DOI:** 10.3390/ijms15022573

**Published:** 2014-02-13

**Authors:** Zhen-Xing Wan, Dong-Mei Yuan, Yi-Ming Zhuo, Xin Yi, Ji Zhou, Zao-Xu Xu, Jian-Lin Zhou

**Affiliations:** Key Laboratory of Protein Chemistry and Developmental Biology of Ministry of Education, College of Life Science, Hunan Normal University, Changsha 410081, China; E-Mails: wanzhenxing88@gmail.com (Z.-X.W.); ydmumina@gmail.com (D.-M.Y.); zhuo715467375@gmail.com (Y.-M.Z.); lefewy.yixin@gmail.com (X.Y.); zhoujihnu@gmail.com (J.Z.); xuzaoxu@gmail.com (Z.-X.X.)

**Keywords:** PA28γ, p53, promoter, transcription regulation, PSME3, negative feedback regulation

## Abstract

PA28γ (also called REGγ, 11Sγ or PSME3) negatively regulates p53 activity by promoting its nuclear export and/or degradation. Here, using the RNA ligase-mediated rapid amplification of cDNA ends (RLM-RACE) method, we identified the transcription start site of the *PA28γ* gene. Assessment with the luciferase assay demonstrated that the sequence −193 to +16 is the basal promoter. Three p53 binding sites were found within the *PA28γ* promoter utilizing a bioinformatics approach and were confirmed by chromatin immunoprecipitation and biotinylated DNA affinity precipitation experiments. The p53 protein promotes *PA28γ* transcription, and p53-stimulated transcription of *PA28γ* can be inhibited by PA28γ itself. Our results suggest that PA28γ and p53 form a negative feedback loop, which maintains the balance of p53 and PA28γ in cells.

## 1. Introduction

The proteasome is a multi-subunit proteolytic complex containing a cylindrical 20S catalytic core particle (20S proteasome) and two regulatory particles (proteasomal activator). There are three classes of proteasomal activators: PA700, PA28, and PA200. PA700 (also called 19S activator), the most common activator, binds to the 20S proteasome to form a 26S proteasome that recognizes and degrades polyubiquitinated proteins in an ATP-dependent manner. PA28 (also called 11S activator, REG) and PA200 activate the ubiquitin- and ATP-independent proteolytic function of the 20S proteasome (reviewed by Tanaka [1]). Of the three PA28 family members, PA28α and PA28β form hetero-oligomers and mainly participate in major histocompatibility complex class I (MHC I) antigen presentation, whereas PA28γ (also called REGγ, 11Sγ, or PSME3) exists as homo-oligomers and is implicated in the regulation of cell proliferation, apoptosis, and carcinogenesis (reviewed by Tanaka and Mao *et al*. [1,2]).

PA28γ has a broad expression pattern, and is especially highly expressed in the nervous system, reproductive system, and cells with proliferative capacity [3]. PA28γ-deficient mice show a slow rate of cell proliferation and small body size [4]. Furthermore, PA28γ has been reported to target the degradation of the cell cycle inhibitors p21^Cip1^, p16^INK4a^ and p19^Arf^ in an ubiquitin-independent manner [5–7]. It also inhibits the activity of the tumor suppressor p53 by promoting its nuclear export and mouse double minute 2 homolog (MDM2)-mediated degradation [8,9]. Moreover, PA28γ has been found to be abnormally expressed in colorectal [10], thyroid [11], breast [12], and laryngeal [13] cancers. These results suggest that *PA28γ* functions as an oncogene. Nonetheless, PA28γ can also play tumor suppressive roles. It has been shown to promote the degradation of oncogenic proteins, such as steroid receptor coactivator-3 [14], hepatitis C virus core protein [15], and PTTG1 [16]. Recent studies demonstrate that PA28γ is required for DNA repair and chromosomal stability [17,18]. PA28γ depletion leads to cellular radiomimetic sensitivity and a significant delay in DNA double-strand break repair [17]. Cells with a depleted expression of *PA28γ* or over-expression of a dominant-negative *PA28γ* mutant demonstrate a marked aneuploidy, supernumerary centrosomes, and multipolarity [18].

Collectively, the information described above suggests that PA28γ exhibits both tumor-promoting and tumor-inhibiting functions in a context-dependent manner. Thus, the precise regulation of *PA28γ* gene expression is important for normal cell function. However, the regulation of *PA28γ* expression has not been elucidated. As a first step to understand this regulation, we identified the transcription start site (TSS) of the gene encoding PA28γ and characterized its promoter. Moreover, we found that the *PA28γ* gene is up-regulated by the tumor suppressor p53 and that its p53-stimulated transcription is inhibited by PA28γ itself.

## 2. Results

### 2.1. Determination of the Transcription Start Site (TSS) of the Human *PA28γ* Gene

**Figure 1.**
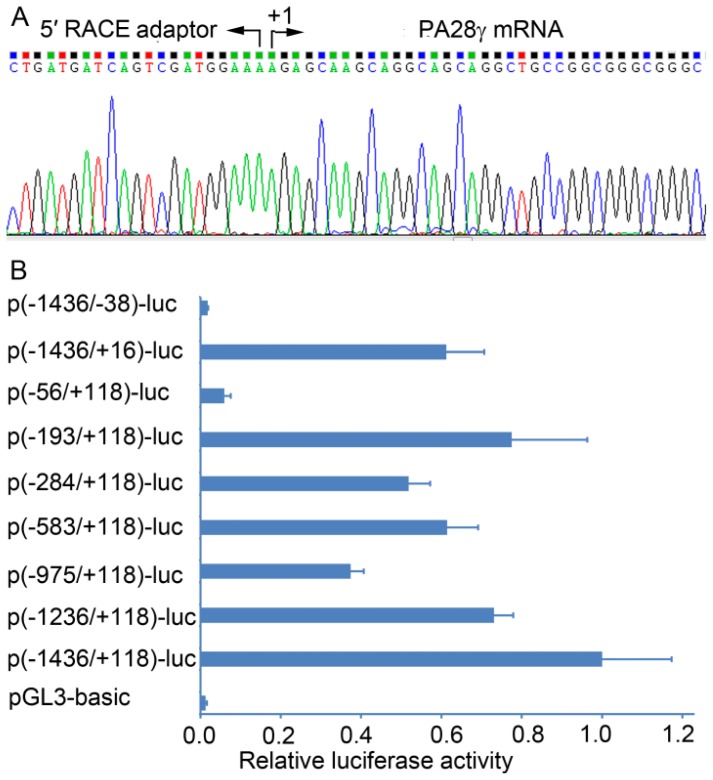
Identification of the transcription start site (TSS) and basal promoter of the human *PA28γ* gene. (**A**) Partial sequencing chromatogram of the 5′ RACE products. Total RNA from HEK293 cells was treated sequentially with calf intestine alkaline phosphatase and tobacco acid pyrophosphatase, and then ligated to the 5′ RACE adaptor. The ligated RNA was reverse transcribed into cDNA and amplified. The final PCR products were cloned into the pMD18-T vector and sequenced; (**B**) Plasmids containing sequentially deleted fragments of the putative PA28γ promoter (−1436 to +118) were transfected into HEK293 cells. Luciferase activity was measured at 36 h post-transfection. The data (means ± S.D.) are represented as the percentage activity relative to that observed in p(−1436/+118)-luc. RACE: rapid amplification of cDNA ends.

A series of computational programs was used to predict the possible TSS of the human *PA28γ* gene. The sites predicted by FPROM [19], TSSW [20], NNPP [21], and McPromoter [22] are located at 497, 193, 127, and 112 nt upstream of the translation start codon, respectively. According to the above results, we designed primers and performed RNA ligase-mediated-rapid amplification of cDNA ends (RLM-RACE) to further determine the exact TSS. This method only reverse transcribes intact mRNAs with a 5′-cap structure [23]. Sequencing the RACE-PCR products revealed that the TSS was adenine (Figure 1A), corresponding with the position 192 nt upstream of the translation start codon.

### 2.2. Identification of the Basal Promoter of the Human *PA28γ* Gene

A 1554 bp segment of the putative promoter sequence (−1436 to +118 relative to the TSS) was cloned into the luciferase reporter vector pGL3-basic, and the generated construct p(−1436/+118)-luc was used as a template to obtain a series of promoter deletion mutants. Each construct was co-transfected with the control plasmid pCMV-LacZ into HEK293 cells, and their luciferase activities were determined at 36 h post-transfection (Figure 1B). The longest construct p(−1436/+118)-luc displayed the highest luciferase activity. The luciferase activities of the deletion constructs p(−1236/+118)-luc and p(−975/+118)-luc were 73% and 37%, respectively, relative to the p(−1436/+118)-luc construct. However, deletion of the sequence from −975 to −583 resulted in a significant increase in the luciferase activity. These results suggested that positive regulatory sites were located in the region between −1436 and −975, and negative regulatory sites were in the region between −975 and −583. The activities of the 5′-unidirectional deletion construct p(−193/+118)-luc and the 3′-unidirectional deletion construct p(−1436/+16) were still much higher than the activity of the vector without promoter pGL3-basic, but the 5′-unidirectional deletion extending from −193 to −56 and the 3′-unidirectional deletion extending from +16 to −38 dramatically decreased the luciferase activities, indicating that the sequence from −193 to +16 is essential for the basal promoter activity of the *PA28γ* gene.

### 2.3. p53 Binds to the Promoter of the Human *PA28γ* Gene

**Figure 2.**
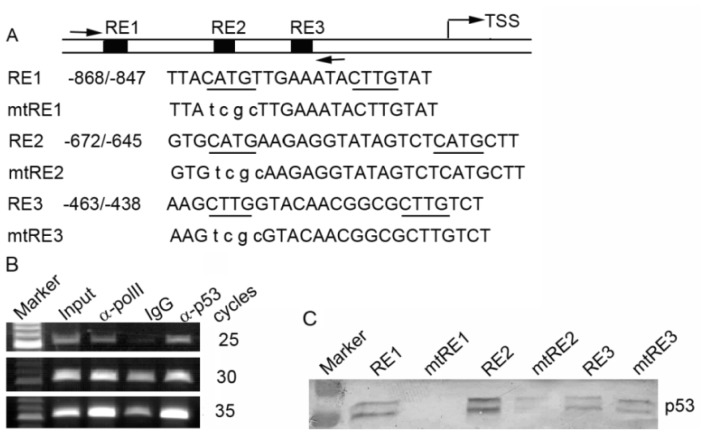
The p53 protein can bind to the promoter of the *PA28γ* gene *in vivo* and *in vitro*. (**A**) Schematic diagram of the *PA28γ* promoter containing three p53 response elements (p53REs). Arrows represent the primers used for chromatin immunoprecipitation (ChIP) PCR. The sequences of wild-type p53REs and corresponding mutants are shown below. The consensus sequences are underlined; (**B**) ChIP was performed in HEK293 cells using anti-p53, anti-RNA polymerase II (positive control), and normal IgG (negative control). DNA from the ChIP was analyzed by semi-quantitative reverse transcription polymerase chain reaction (RT-PCR) in triplicate with three different PCR cycle numbers (25, 30 and 35); (**C**) The recombinant p53 protein was incubated with biotinylated wild-type or mutant RE oligonucleotides, and the DNA/protein complex was pulled down with streptavidin-agarose beads. The amount of bound p53 protein was detected by western blotting.

To explore what transcription factors contribute to the regulation of the *PA28γ* gene, we used bioinformatics tools (ConTra [24] and MatInspector [25]) to search the promoter region of *PA28γ* for potential transcription factor binding sites and identified three p53 response elements (p53REs): RE1 (nucleotides −868 to −847), RE2 (nucleotides −672 to −645), and RE3 (nucleotides −463 to −438) (Figure 2A). To determine whether p53 can bind to these p53REs *in vivo*, we performed chromatin immunoprecipitation (ChIP) in HEK293 cells using anti-p53 antibody, anti-RNA polII antibody, or normal mouse IgG. The immunoprecipitated DNA was analyzed by semi-quantitative reverse transcription polymerase chain reaction (RT-PCR) with primers corresponding to the region (−933 to −407) containing the three potential p53REs. As shown in Figure 2B, a PCR product was observed in the ChIP with anti-p53 and anti-RNA polII, but substantially less was detected in the normal rabbit IgG ChIP, suggesting that p53 can bind to the *PA28γ* promoter *in vivo*. To further confirm the binding of p53 and the potential p53REs, we used biotinylated double-stranded oligonucleotides containing wild-type or mutant p53RE to precipitate recombinant p53 protein. As shown in Figure 2C, the RE1 and RE2 oligonucleotides containing wild-type p53RE can precipitate the p53 protein, but their corresponding mutant counterparts cannot. The wild-type and mutant RE3 have weak affinities for the p53 protein. These results indicate that p53 can bind to the promoter of the human *PA28γ* gene both *in vivo* and *in vitro*.

### 2.4. p53 Stimulates the Transcription of Human *PA28γ*

**Figure 3.**
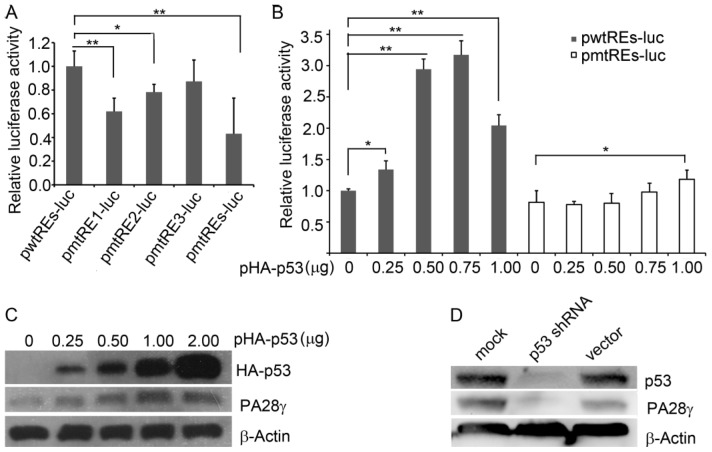
The p53 protein stimulates the expression of the human *PA28γ* gene. (**A**) HEK293 cells were transfected with pwtREs-luc and its mutant plasmids, or (**B**) co-transfected with pwtREs-luc (or pmtREs-luc) and pHA-p53. Luciferase activity was measured at 36 h post-transfection. The data are presented as means ± S.D. of triplicate samples. * *p <* 0.05, ** *p <* 0.01; (**C**) HEK293 cells were transfected with p53 expression plasmid pHA-p53, while (**D**) the HEK293 cells were transduced with lentivirus expressing p53 shRNA. The cells transfected with plasmids were harvested at 36 h post-transfection, while the cells transduced with lentivirus were harvested at 72 h post-transduction. The protein levels of the HA-p53 fusion protein, endogenous p53, PA28γ, and β-actin were detected by western blotting.

To examine the functional significance of p53 binding, we cloned the promoter region (−975 to −407) containing the three p53REs (Figure 2A) into the pTAL-Luc vector. The generated construct pwtREs-luc and its mutant constructs (pmtRE1-luc, pmtRE2-luc, pmtRE3-luc, and pmtREs-luc representing mutations of RE1, RE2, RE3 and all three REs, respectively) were subsequently transfected into HEK293 cells. Compared with pwtREs-luc, mutation of RE1, RE2, or the three REs significantly decreased the luciferase activity. Consistent with the above DNA binding assay (Figure 2C), mutation of RE3 only slightly affected the luciferase activity (Figure 3A). To further investigate the effect of p53, we co-transfected increasing amounts of the p53 expression plasmid and a constant amount of pwtREs-luc (with three wild-type p53REs) or pmtREs-luc (with three mutant p53REs) into HEK293 cells. The luciferase assay showed that the luciferase activity of the cells transfected with plasmid pwtREs-luc increased with increasing amounts of p53 plasmid (0 to 0.75 μg), but that the luciferase activity decreased as the amount of p53 plasmid increased to 1 μg. However, with increasing p53 expression, the increase in luciferase activity in cells expressing pmtREs-luc was significantly less than in cells expressing pwtREs-luc (Figure 3B).

To determine the influence of p53 on the endogenous PA28γ levels, cells were transfected with the p53 expression plasmid or p53 shRNA lentivirus and the level of endogenous PA28γ protein was determined. The over-expression of p53 in HEK293 cells increased PA28γ protein levels (Figure 3C), whereas the knockdown of p53 in HEK293 cells decreased PA28γ protein levels (Figure 3D). In accordance with the luciferase assay (Figure 3B), PA28γ protein levels began to decrease when the amount of the p53 plasmid approached 2 μg. These findings suggest that PA28γ has a negative feedback effect on itself.

### 2.5. p53-Induced Transcription of *PA28γ* Is Suppressed by PA28γ Itself

**Figure 4.**
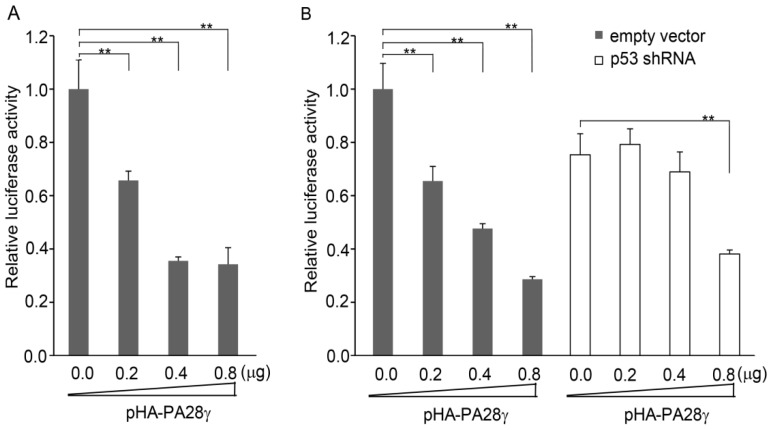
The activity of the *PA28γ* promoter is suppressed by PA28γ itself. (**A**) The plasmid pwtREs-luc, containing 3 p53 response elements (p53Res) was co-transfected into HEK293 cells with increasing amounts of the PA28γ expression construct pHA-PA28γ; (**B**) The full-length *PA28γ* promoter construct p(−1436/+118)-luc was co-transfected with increasing amounts of the *PA28γ* expression construct pHA-PA28 into cells, which were previously transduced with either lentivirus expressing p53 shRNA or control lentivirus. Luciferase activity was measured at 36 h post-transfection. The data (means ± S.D.) are presented as the percentage activity relative to that observed in cells without pHA-PA28γ. The experiments were performed in triplicate. * *p* < 0.05, ** *p <* 0.01.

PA28γ has previously been reported to inhibit the activity of p53 by facilitating its nuclear export and degradation [8,9]. Therefore, we investigated whether the p53-stimulated transcription of *PA28γ* can be repressed by PA28γ itself. First, we transfected the p53-responsive reporter pwtREs-luc and PA28γ expression construct pHA-PA28γ into HEK293 cells. The luciferase assay showed that with an increase in *PA28γ* expression, the luciferase activity decreased (Figure 4A), indicating that PA28γ suppresses the activity of p53.

Subsequently, we transfected the *PA28γ* promoter reporter construct p(−1436/+118)-luc into HEK293 cells that had been transduced with either lentivirus expressing p53 shRNA or control lentivirus. In HEK293 cells transduced with the control lentivirus, the over-expression of *PA28γ* inhibited the activity of the PA28γ promoter, but had no significant effect on HEK293 cells in which p53 expression was knocked down by the p53 shRNA lentivirus (Figure 4B).

Taken together, the above results suggested that PA28γ inhibits the transcription of *PA28γ* itself by suppressing the activity of p53.

## 3. Discussion

PA28γ has been shown to be a negative regulator of p53 activity [8,9]. Interestingly, we were able to demonstrate that the p53 protein is able to bind to the promoter of the *PA28γ* gene and up-regulate its expression. Moreover, the p53-stimulated transcription of *PA28γ* can be suppressed by PA28γ itself. These results suggest that PA28γ and p53 form a negative feedback loop. This reciprocal feedback regulation of PA28γ and p53 is similar to that of MDM2 and p53, in which the increase in p53 leads to an increase in *Mdm2* transcription and MDM2 targets p53 for degradation [26]. Actually, PA28γ exerts its role of feedback regulation of p53 in an MDM2-dependent manner. PA28γ enhances the interaction of MDM2 and p53 and promotes the MDM2-mediated mono-ubiquitylation and/or multi-ubiquitylation of p53, leading to its nuclear export and/or degradation [8,9]. We propose that PA28γ may function as a cofactor of MDM2 in maintaining an appropriate level of p53.

The reciprocal feedback regulation between PA28γ and p53 also helps to maintain the expression of PA28γ in cells at the appropriate level. When the PA28γ protein accumulates in a cell harboring p53, it inactivates p53 function, and this inactivation of p53 results in a decrease in *PA28γ* transcription. Many studies have demonstrated that maintaining a proper level of PA28γ is crucial because both the over-expression and under-expression of the *PA28γ* gene could adversely affect normal cell function. For example, *PA28γ* is abnormally expressed in several cancers, such as colorectal [10], thyroid [11], and breast cancers [12], and its over-expression promotes the proliferation and growth of cancer cells. However, PA28γ is required for normal cell function, and the loss of PA28γ leads to the failure of DNA double-strand break repair [17] and chromosomal instability [18].

## 4. Materials and Methods

### 4.1. Plasmid Constructs

The putative promoter (nucleotides −1436 to +118 relative to the TSS) of *PA28γ* was amplified from human genomic DNA and inserted into the promoterless firefly luciferase reporter vector pGL3-Basic (Promega, Madison, WI, USA), and the resulting construct was denoted p(−1436/+118)-luc. Subsequently, a series of promoter deletion constructs was generated by PCR using p(−1436/+118)-luc as a template. A fragment of the *PA28γ* promoter (nucleotides −975 to −407) containing three putative p53 response elements (REs) was inserted upstream of the minimal TATA-like promoter in the firefly luciferase reporter vector pTAL-Luc (Clontech, Mountain View, CA, USA), and the resulting construct was denoted pwtREs-Luc. Site-directed mutagenesis of the construct pwtREs-Luc was performed by overlapping PCR. To generate the PA28γ expression construct pHA-PA28γ, the coding region of human *PA28γ* was cloned in-frame with Myc-tag into pCMV-HA. The HA-tagged p53 expression plasmid pHA-p53 was described previously [27].

### 4.2. Computational Analysis of the Promoter

The transcription start site of the human *PA28γ* gene was predicted by online bioinformatics tools: NNPP [21] (http://www.fruitfly.org/seq_tools/promoter.html), McPromoter [22] (http://tools.igsp.duke.edu/generegulation/McPromoterMMII/), and Softberry programs FPROM [19]/TSSW [20] (http://linux1.softberry.com/berry.phtml). ConTra [24] (http://bioit.dmbr.ugent.be/ConTra/index.php) and MatInspector [25] (http://www.genomatix.de) were used to search for the potential transcription factor sites within the promoter.

### 4.3. Cell Culture and Transfection

Human embryonic kidney-293 (HEK293) cells (ATCC, Manassas, VA, USA) were cultured in DMEM media, supplemented with glutamine, antibiotics, and 10% fetal bovine serum at 37 °C and 5% CO_2_. Transfection was performed using Lipofectamine 2000 (Invitrogen, Carlsbad, CA, USA) according to the manufacturer’s instructions.

### 4.4. Luciferase Assay

Each firefly luciferase reporter construct was co-transfected with pCMV-lacZ (Clontech, Palo Alto, CA, USA) encoding β-galactosidase, which serves as an internal control to normalize the transfection efficiency. At 36 h after transfection, cells were lysed to measure the luciferase and β-galactosidase activities, as previously described [27]. Luciferase activity was normalized to β-galactosidase activity.

### 4.5. RLM-RACE Assay

RLM-RACE was performed using the 5′-Full RACE Kit (TaKaRa, Dalian, China) according to the manufacturer’s instructions. Briefly, 1 μg of total RNA from HEK293 cells was treated sequentially with calf intestine alkaline phosphatase and tobacco acid pyrophosphatase, and then ligated into the 5′-RACE adaptor. The ligated RNA was reverse transcribed into cDNA using M-MLV reverse transcriptase and a random 9-mer primer. The cDNA was used as a template for PCR with a gene-specific outer primer and the 5′ RACE outer primer, followed by a nested PCR (Eppendorf, Hamburg, Germany) using a gene-specific inner primer and the 5′ RACE inner primer. The final PCR products were cloned into a pMD18-T vector (TaKaRa, Dalian, China) and sequenced. The 5′ RACE adaptor, 5′ RACE outer primer and 5′-RACE inner primer were provided in the kit. The *PA28γ* gene-specific primers were as follows: 5′-GTCAGGGACTGGGAGATTCA-3′ (outer) and 5′-CCACCAAGTCTTCTGCCTCACT-3′ (inner).

### 4.6. ChIP Assay

The ChIP assay was performed using EZ-ChIP kit (Millipore, Temecula, CA, USA) according to the manufacturer’s instructions. HEK293 cells were cultured to approximately 90% confluency in a 10 cm plate and then cross-linked in 1% formaldehyde for 10 min at room temperature. Cross-linking was stopped by the addition of 1 M glycine to a final concentration of 125 mM for 5 min. The cells were subsequently washed with PBS and PBS containing 0.5% protease inhibitor cocktail. The cell lysate was prepared using SDS lysis buffer (1% SDS, 10 mM EDTA, 50 mM Tris, pH 8.1), and sheared using a sonicator until DNA fragments of 200–1000 bp were formed. The crosslinked chromatin was precipitated using anti-p53 (ChIPAb+ p53, Millipore, Temecula, CA, USA), mouse normal IgG (negative control) or anti-RNA polymerase II (positive control). Protein/DNA complexes were reverse cross-linked and the DNA fragments were purified. The amount of precipitated DNA was detected by semi-quantitative RT-PCR; the PCR cycles for linear amplification had been selected in preliminary experiments. The final PCR products were separated by electrophoresis on a 1.5% agarose gel and visualized by ethidium bromide. The primers used were 5′-CGCACTGGATTTTGAAGACTT-3′ and 5′-CGAGGCTCAAGTGTTTAGGC-3′.

### 4.7. Biotinylated DNA Affinity Precipitation

DNA binding activity was detected by biotinylated DNA affinity precipitation as described by Zhang H. *et al.* [28]. Briefly, 20 μg purified p53 recombinant protein was dissolved in gel shift binding buffer (final concentration: 10 mM Tris–HCl, pH 8.0, 40 mM KCl, 1 mM dithiothreitol (DTT), 6% glycerol, and 0.05% NP-40), precleared with 50 μL of streptavidin-coated agarose beads (Pierce, Rockford, IL, USA), and incubated with 1 μg of biotinylated oligonucleotides overnight at 4 °C under gentle rocking. Fifty microliters of streptavidin-coated agarose beads (50% slurry) was added and the mixture was incubated for another 2 h. The beads containing bound complexes were recovered by centrifugation at 1000–2000 rpm for 1 min at 4 °C and washed sequentially with Tris–EDTA (100 mM NaCl), gel shift binding buffer, and PBS. The protein was eluted from the beads with 40 μL of sample loading buffer by heating at 95–100 °C for 5 min and subsequently separated by 12% SDS-PAGE. The amount of bound p53 protein was detected by western blotting.

### 4.8. RNA Interference

HEK293 cells were transduced with either lentivirus-expressing p53 shRNA or control lentivirus (all obtained from GeneChem, Shanghai, China) according to the manufacturer’s instructions. At 72 h after transduction, the cell lysate was collected and analyzed by western blotting for the expression of p53 and PA28γ.

### 4.9. Statistical Analysis

Microsoft Excel was used for the statistical analysis. Student’s *t*-test was performed to evaluate the significance of the differences between samples.

## 5. Conclusions

In conclusion, we have characterized the promoter of *PA28γ* and demonstrated that p53 promotes the expression of its negative regulator, PA28γ. Our results suggest that PA28γ and p53 form a negative feedback loop that maintains the balance of p53 and PA28γ in the cells.

## References

[1] Tanaka K. (2009). The proteasome: Overview of structure and functions. Proc. Jpn. Acad. Ser. B.

[2] Mao I., Liu J., Li X., Luo H. (2008). REGγ, a proteasome activator and beyond?. Cell. Mol. Life Sci.

[3] Yu G., Zhao Y., He J., Lonard D.M., Mao C.A., Wang G., Li M., Li X. (2010). Comparative analysis of REGγ expression in mouse and human tissues. J. Mol. Cell Biol.

[4] Murata S., Kawahara H., Tohma S., Yamamoto K., Kasahara M., Nabeshima Y., Tanaka K., Chiba T. (1999). Growth retardation in mice lacking the proteasome activator PA28γ. J. Biol. Chem.

[5] Chen X., Barton L.F., Chi Y., Clurman B.E., Roberts J.M. (2007). Ubiquitin-independent degradation of cell-cycle inhibitors by the REGγ proteasome. Mol. Cell.

[6] Li X., Amazit L., Long W., Lonard D.M., Monaco J.J., O’Malley B.W. (2007). Ubiquitin- and ATP-independent proteolytic turnover of p21 by the REGγ-proteasome pathway. Mol. Cell.

[7] Kobayashi T., Wang J., Al-Ahmadie H., Abate-Shen C. (2013). ARF regulates the stability of p16 protein via REGγ-dependent proteasome degradation. Mol. Cancer Res.

[8] Zhang Z., Zhang R. (2008). Proteasome activator PA28γ regulates p53 by enhancing its MDM2-mediated degradation. EMBO J.

[9] Liu J., Yu G., Zhao Y., Zhao D., Wang Y., Wang L., Liu J., Li L., Zeng Y., Dang Y. (2010). REGγ modulates p53 activity by regulating its cellular localization. J. Cell Sci.

[10] Roessler M., Rollinger W., Mantovani-Endl L., Hagmann M.L., Palme S., Berndt P., Engel A.M., Pfeffer M., Karl J., Bodenmüller H. (2006). Identification of PSME3 as a novel serum tumor marker for colorectal cancer by combining two-dimensional polyacrylamide gel electrophoresis with a strictly mass spectrometry-based approach for data analysis. Mol. Cell Proteomics.

[11] Okamura T., Taniguchi S., Ohkura T., Yoshida A., Shimizu H., Sakai M., Maeta H., Fukui H., Ueta Y., Hisatome I. (2003). Abnormally high expression of proteasome activator-γ in thyroid neoplasm. J. Clin. Endocrinol. Metab.

[12] Wang X., Tu S., Tan J., Tian T., Ran L., Rodier J.F., Ren G. (2011). REGγ: A potential marker in breast cancer and effect on cell cycle and proliferation of breast cancer cell. Med. Oncol.

[13] Li L.P., Cheng W.B., Li H., Li W., Yang H., Wen D.H., Tang Y.D. (2012). Expression of proteasome activator REGγ in human laryngeal carcinoma and associations with tumor suppressor proteins. Asian Pac. J. Cancer Prev.

[14] Li X., Lonard D.M., Jung S.Y., Malovannaya A., Feng Q., Qin J., Tsai S.Y., Tsai M.J., O’Malley B.W. (2006). The SRC-3/AIB1 coactivator is degraded in a ubiquitin- and ATP-independent manner by the REGγ proteasome. Cell.

[15] Moriishi1 K., Okabayashi1 T., Nakai1 K., Moriya K., Koike K., Murata S., Chiba T., Tanaka K., Suzuki E., Suzuki T. (2003). Proteasome activator PA28γ-dependent nuclear retention and degradation of hepatitis C virus core protein. J. Virol.

[16] Ying H., Furuya F., Zhao L., Araki O., West B.L., Hanover J.A., Willingham M.C., Cheng S.Y. (2006). Aberrant accumulation of PTTG1 induced by a mutated thyroid hormone β receptor inhibits mitotic progression. J. Clin. Invest.

[17] Levy-Barda A., Lerenthal Y., Davis A.J., Chung Y.M., Essers J., Shao Z., van Vliet N., Chen D.J., Hu M.C.T., Kanaar R. (2011). Involvement of the nuclear proteasome activator PA28γ in the cellular response to DNA double-strand breaks. Cell Cycle.

[18] Zannini L., Lecis D., Buscemi G., Carlessi L., Gasparini P., Fontanella E., Lisanti S., Barton L., Delia D. (2008). REGγ proteasome activator is involved in the maintenance of chromosomal stability. Cell Cycle.

[19] Solovyev V., Kosarev P., Seledsov I., Vorobyev D. (2006). Automatic annotation of eukaryotic genes, pseudogenes and promoters. Genome Biol.

[20] Solovyev V., Salamov A. (1997). The Gene-Finder computer tools for analysis of human and model organisms genome sequences. Proc. Int. Conf. Intell. Syst. Mol. Biol.

[21] Reese M.G. (2001). Application of a time-delay neural network to promoter annotation in the *Drosophila melanogaster* genome. Comput. Chem.

[22] Ohler U., Stemmer G., Harbeck S., Niemann H. (2000). Stochastic segment models of eukaryotic promoter regions. Pac. Symp. Biocomput.

[23] Maruyama K., Sugano S. (1994). Oligo-capping: A simple method to replace the cap structure of eukaryotic mRNAs with oligoribonucleotides. Gene.

[24] Broos S., Hulpiau P., Galle J., Hooghe B., van Roy F., de Bleser P. (2011). ConTra v2: A tool to identify transcription factor binding sites across species, update 2011. Nucleic Acids Res.

[25] Cartharius K., Frech K., Grote K., Klocke B., Haltmeier M., Klingenhoff A., Frisch M., Bayerlein M., Werner T. (2005). MatInspector and beyond: Promoter analysis based on transcription factor binding sites. Bioinformatics.

[26] Wu X., Bayle J.H., Olson D., Levine A.J. (1993). The p53-mdm-2 autoregulatory feedback loop. Genes Dev.

[27] Zhou J., Qiao X., Xiao L., Sun W., Wang L., Li H., Wu Y., Ding X., Hu X., Zhou C. (2010). Identification and characterization of the novel protein CCDC106 that interacts with p53 and promotes its degradation. FEBS Lett.

[28] Zhang H., Chen H., Luo H., An J., Sun L., Mei L., He C., Jiang L., Jiang W., Xia K. (2012). Functional analysis of Waardenburg syndrome-associated PAX3 and SOX10 mutations: Report of a dominant-negative SOX10 mutation in Waardenburg syndrome type II. Hum. Genet.

